# Dysregulation of IL-17/IL-22 Effector Functions in Blood and Gut Mucosal Gamma Delta T Cells Correlates With Increase in Circulating Leaky Gut and Inflammatory Markers During cART-Treated Chronic SIV Infection in Macaques

**DOI:** 10.3389/fimmu.2021.647398

**Published:** 2021-02-25

**Authors:** Edith M. Walker, Nadia Slisarenko, Giovanni L. Gerrets, Brooke F. Grasperge, Julie A. Mattison, Patricia J. Kissinger, David A. Welsh, Ronald S. Veazey, S. Michal Jazwinski, Namita Rout

**Affiliations:** ^1^ Division of Microbiology, Tulane National Primate Research Center, Covington, LA, United States; ^2^ Veterinary Medicine, Tulane National Primate Research Center, Covington, LA, United States; ^3^ Translational Gerontology Branch, National Institute on Aging, NIH, Poolesville, MD, United States; ^4^ School of Public Health & Tropical Medicine, Tulane University, New Orleans, LA, United States; ^5^ Department of Microbiology, Immunology and Parasitology, Louisiana State University School of Medicine, New Orleans, LA, United States; ^6^ Division of Comparative Pathology, Tulane National Primate Research Center, Covington, LA, United States; ^7^ Tulane Center for Aging, Tulane University School of Medicine, New Orleans, LA, United States

**Keywords:** SIV, intestinal epithelial barrier disruption, IFABP, LBP, sCD14, gammadelta T cells

## Abstract

HIV-associated inflammation has been implicated in the premature aging and increased risk of age-associated comorbidities in cART-treated individuals. However, the immune mechanisms underlying the chronic inflammatory state of cART-suppressed HIV infection remain unclear. Here, we investigated the role of γδT cells, a group of innate IL-17 producing T lymphocytes, in the development of systemic inflammation and leaky gut phenotype during cART-suppressed SIV infection of macaques. Plasma levels of inflammatory mediators, intestinal epithelial barrier disruption (IEBD) and microbial translocation (MT) biomarkers, and Th1/Th17-type cytokine functions were longitudinally assessed in blood and gut mucosa of SIV-infected, cART-suppressed macaques. Among the various gut mucosal IL-17/IL-22-producing T lymphocyte subsets including Th17, γδT, CD161^+^ CD8^+^ T, and MAIT cells, a specific decline in the Vδ2 subset of γδT cells and impaired IL-17/IL-22 production in γδT cells significantly correlated with the subsequent increase in plasma IEBD/MT markers (IFABP, LPS-binding protein, and sCD14) and pro-inflammatory cytokines (IL-6, IL-1β, IP10, etc.) despite continued viral suppression during long-term cART. Further, the plasma inflammatory cytokine signature during long-term cART was distinct from acute SIV infection and resembled the inflammatory cytokine profile of uninfected aging (inflammaging) macaques. Overall, our data suggest that during cART-suppressed chronic SIV infection, dysregulation of IL-17/IL-22 cytokine effector functions and decline of Vδ2 γδT cell subsets may contribute to gut epithelial barrier disruption and development of a distinct plasma inflammatory signature characteristic of inflammaging. Our results advance the current understanding of the impact of chronic HIV/SIV infection on γδT cell functions and demonstrate that in the setting of long-term cART, the loss of epithelial barrier-protective functions of Vδ2 T cells and ensuing IEBD/MT occurs before the hallmark expansion of Vδ1 subsets and skewed Vδ2/Vδ1 ratio. Thus, our work suggests that novel therapeutic approaches toward restoring IL-17/IL-22 cytokine functions of intestinal Vδ2 T cells may be beneficial in preserving gut epithelial barrier function and reducing chronic inflammation in HIV-infected individuals.

## Introduction

The discovery of efficient, well-tolerated combinational antiretroviral therapy (cART) regimens has remarkably reduced the rates of HIV-associated mortality and transformed it into a chronic, manageable disease that requires life-long treatment. However, people living with HIV (PLWH) still remain at unusually high risk of age-associated diseases including neurocognitive, metabolic, cardiovascular disorders, and other non-AIDS-defining comorbidities (1). The pathogenesis of age-associated diseases is complex but there is growing consensus on the substantial role of HIV-associated chronic inflammation and immune activation in the premature onset of immunosenescence and early aging in PLWH despite effective viral suppression (2–4). A similar process characterized by increased pro-inflammatory mediators leading to a chronic inflammatory state, termed “inflammaging”, is a significant risk factor for morbidity and mortality in the elderly people, even those without HIV infection (5). As more PLWH are transitioning to the middle-age and older age-groups, it is important to develop a detailed understanding of the immune mechanisms underlying HIV-associated inflammaging and determine the immune mechanisms that may synergize with aging and may accelerate this process.

Aberrant immune activation and chronic inflammation during HIV/SIV infection is strongly associated with loss of Th17-type mucosal immune functions and intestinal epithelial barrier damage (IEBD) resulting in intestinal permeability (leaky gut) and microbial translocation (MT) (6–9). This leaky gut-associated chronic inflammation persists even with long-term effective cART and predicts mortality and incidence of age-related co-morbidities (e.g. neurocognitive, metabolic, cardiovascular disorders) (10–12). Accordingly, an inflammaging phenotype comprising of IEBD, MT, and inflammatory biomarkers has been described in both HIV-negative older subjects and PLWH (8). Indeed, we have recently demonstrated a similar inflammaging phenotype in SIV-negative aging rhesus macaques with elevated plasma levels of IEBD, MT, and inflammatory markers, which is associated with significant impairment in production of the cytokines IL-17 and IL-22 by classical and innate Th17-type immune cells (13). Gamma delta (γδ) T cells are an important innate source of IL-17 and IL-22 cytokines and are key players in gut barrier functions, MT, and immune activation during HIV infection (14), and may demonstrate both inflammatory (15, 16) and/or immuno-regulatory potential (17, 18). HIV infection results in changes in circulating γδ T cell subsets with an increase in Vδ1 and depletion of Vδ2 T cells that may persist with viral suppression (19, 20). Similar expansion in Vδ1 T cells during chronic untreated SIV infection has been associated with MT (21). However, the dynamic role of gut mucosal γδ T cells in IEBD, MT and persistent inflammation through the course of treated HIV infection remains unclear.

Here we assessed the development of systemic inflammation during chronic SIV infection suppressed with cART in the rhesus macaque model of treated HIV infection and evaluated longitudinal changes in systemic and mucosal γδ T cell functions. We demonstrate that following early resolution of circulating inflammatory markers by effective viral suppression with cART, a plasma inflammatory and leaky gut phenotype similar to inflammaging re-emerged during later stages of chronic SIV infection despite continued treatment and viral suppression. The development of this phenotype was preceded by a significant loss of systemic and gut mucosal IL-17/IL-22 cytokine producing functions of γδT cells along with a significant decline in Vδ2 cells and subsequent increase in Vδ1 cells resulting in skewing of the Vδ2/Vδ1 subset ratio during long-term suppressive ART. These data are the first to demonstrate that dysregulation of γδT cells, particularly Vδ2 T cells in the gut mucosa, is likely a cause rather than an effect of leaky gut and systemic inflammation during chronic treated SIV infection.

## Materials and Methods

### Ethics Statement

Animals in this study were housed at the Tulane National Primate Research Center (TNPRC), accredited by the Association for Assessment and Accreditation of Laboratory Animal Care (AAALAC) International. The study was approved by the TNPRC Institutional Animal Care and Use Committee (IACUC) and was conducted under the standards of the US National Institutes of Health Guide for the Care and Use of Laboratory Animals. Following SIV infection, animals were housed in Animal Biosafety Level 2 indoor housing. All animal procedures including virus administration, sample collection, and euthanasia were carried out under the direction of TNPRC veterinarians.

### Animals, Viral Inoculation, and cART

Six healthy female Indian origin rhesus macaques ranging in age from 5–10 years old and seronegative for SIV, HIV-2, STLV-1 (Simian T Leukemia Virus type-1), SRV-1 (type D retrovirus), and herpes-B viruses were used in this study. MHC-1 genotyping for exclusion of the common Mamu alleles Mamu-A*01/-A*02 and Mamu-B*08/-B*17 was performed by sequence-specific priming PCR. Animals were infected with 2500 TCID50 SIV_mac_251 *via* intrarectal (IR) route using the pathogenic SIV challenge stocks obtained from the Preclinical Research and Development Branch of Vaccine and Prevention Research Program, Division of AIDS, NIAID. cART consisted of daily subcutaneous injection of 5.1 mg/kg Tenofovir Disoproxil Fumarate (TDF), 30 mg/kg Emtricitabine (FTC) and 2.5 mg/kg Dolutegravir (DTG) in a solution containing 15% (v/v) kleptose at pH 4.2, as previously described (22).

### Sample Collection and Processing

Blood samples in EDTA vacutainer tubes (Sarstedt Inc Newton, NC) were taken for a complete blood count and routine chemical analysis, and centrifuged within 1 h of phlebotomy for plasma separation. Processing of blood and rectal (RB) biopsies were performed as previously described for isolation of PBMC and lamina propria lymphocytes (23). Plasma viral load quantification was performed using Roche High Pure Viral RNA Kit (Catalog #11858882001) as earlier described (24).

### Plasma Markers of Inflammation, Microbial Translocation, and Intestinal Damage

Frozen plasma samples were thawed and cleared using Ultrafree Centrifugal Filters (Millipore, Billerica, MA). The filtered plasma samples were used for simultaneous quantification of cytokines, chemokines, and growth factors using the multiplexed-bead assay Non-Human Primate Cytokine & Chemokine & Growth Factor 37-plex ProcartaPlex (Invitrogen, Life Technologies), following manufacturers’ instructions. Data were acquired with a Bio- Plex 200 analyzer (Bio-Rad, Hercules, CA) and analyzed using Bio-Plex Manager software v6.1 (BioRad). For the analysis of markers of leaky gut and microbial translocation, plasma IFABP and LBP were quantified using the commercially available Monkey IFABP/FABP2 and LBP ELISA kits (MyBioSource, San Diego, CA). Commercially available ELISA kit for Human sCD14 (R&D Systems, Minneapolis, MN) was used according to the manufacturer’s protocols with 1:200 diluted plasma samples. All tests were performed according to the manufacturer’s guidelines. The assays were performed in duplicate, and data were analyzed using Gen 5 software (BioTek).

### Flow Cytometric Analysis

Multi-color flowcytometric analysis was performed on cells according to standard procedures using anti-human mAbs that cross-react with rhesus macaques. The following antibodies were used at predetermined optimal concentrations: CD45 (BD clone D058-1283), CD3 APC-Cy7 (BD clone SP34-2), CD4 BV605 (BD clone L200), CD8 BV650 (BD clone SK1), CD69 PE-CF594 (BD clone FN50), CD161 PE (Biolegend clone HP- 3G10), TCR γδ PE-Cy7 (BD clone B1), TCR Vδ1 FITC (ThermoScientific clone TS8.2), TCR Vδ2 FITC (ThermoScientific clone 15D), TCR Vα7.2 BV421 (Biolegend clone 3C10), and Aqua Live/Dead amine dye-AmCyan from Invitrogen (Waltham, MA). Surface staining was carried out by standard procedures as earlier described (23). Flow cytometric acquisition was performed on the BD Fortessa instrument driven by the FACS DiVa software for at least 100,000 CD3+ T cells in PBMC or at least 10,000 CD3+ T cells for rectal biopsy lymphocytes. The data acquired were analyzed using FlowJo software (version 10.7.1; TreeStar, Ashland, OR). For evaluation of cytokine production, intracellular cytokine staining with IFN-γ BV510 (Biolegend clone 4S.B3), IL-17 PerCP-Cy5.5 (eBioscience clone eBio64DEC17), IL-22 APC (Invitrogen clone IL22JOP), and TNF-α Alexa Fluor 700 (BD clone MAb11) were utilized.

### Intracellular Cytokine Staining

Freshly isolated PBMCs and rectal biopsy lamina propria lymphocytes were resuspended at a concentration of 1 × 10^6^ cells per ml in RPMI 1640 medium supplemented with 10% FBS, 100 IU ml^−1^ penicillin, and 100 μg ml^−1^ streptomycin. Stimulations were conducted for 16 h at 37 °C in the presence of phorbol myristate acetate (PMA; 10 ng ml^−1^), ionomycin (Sigma-Aldrich 1 μg ml^−1^), in the presence of brefeldin A (5 μg/ml, Sigma-Aldrich). After 16 h, cells were washed once with PBS to remove stimuli and stained with surface markers for CD45, CD3, CD4, CD8, TCR γδ, TCR Vδ1, or TCR Vδ2, and CD161 for 30 min at room temperature. Cells were then fixed with cytofix/cytoperm (BD Pharmingen, San Diego, CA), washed, and stained intracellularly with antibodies specific for CD69, IL-17, IL-22, TNF-α, and IFN-γ for 30 min at room temperature. Following staining, cells were washed and fixed with PBS containing 1% paraformaldehyde prior to acquiring on BD Fortessa cytometer.

### Statistical Analyses

All statistical analysis was performed using GraphPad Prism Software (Version 8.4.3). Data were analyzed by one-way analysis of variance (ANOVA) with multiple comparisons, one-way ANOVA with a test for linear trend, or two-way ANOVA with repeated measures. Tukey’s and Dunnett’s *post hoc* tests were used for multiple comparisons. All correlations were computed using non-parametric Spearman rank correlation test. *P* values of 0.05 or lower were considered significant, ^∗^p<0.05, ^∗∗^p<0.01, ^∗∗∗^p<0.001, ^∗∗∗∗^p<0.0001. Polyfunctional responses were compared using SPICE 6 software (25).

## Results

### Early Resolution of SIV-Induced Increase in Inflammatory Cytokines and IEBD Biomarkers Following Viral Suppression With cART

**Figure 1:**
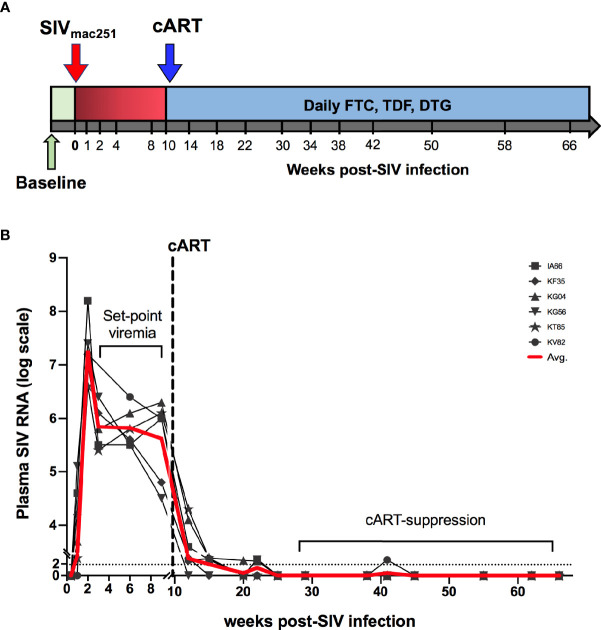
Study schedule and plasma viral loads. **(A)** Animals (n = 6 per group) were treated with cART consisting of daily subcutaneous injection of Tenofovir Disoproxil Fumarate (TDF), Emtricitabine (FTC) and Dolutegravir (DTG). Two sets of baseline samples were collected prior to SIV infection on d0 and cART was started 10 weeks following SIV infection. **(B)** Longitudinal SIV RNA quantification in plasma is shown for the first 75 weeks of the study. Dashed line represents the beginning of cART. Red line is the average viral load for all animals.

Six adult rhesus macaques were infected *via* intra-rectal route with SIVmac251 to represent mucosal HIV infection. Following establishment of set-point viremia, all animals were treated daily with a three-drug antiretroviral (cART) regimen, Tenofovir Disoproxil Fumarate (TDF), Emtricitabine (FTC) and Dolutegravir (DTG) (as indicated by the blue shading in **Figure 1A**). As expected, plasma viremia peaked at 2 weeks of infection to an average 7.2 log_10_ copies/mL and by 4 weeks had reduced 2 logs and settled at ~5.8 log_10_ copies/mL (**Figure 1B**). Stable suppression of viremia below the limit of detection (LOD) of the assay (83 viral copy Eq/ml) was achieved in all 6 animals 8 weeks from cART initiation (18 weeks post-SIV infection). To assess the impact of SIV infection on systemic inflammation, plasma concentrations of inflammatory and IEBD biomarkers were measured at pre-infection (2–4 weeks before SIV infection), around peak viremia (1–2 weeks post-SIV), during early cART (1–6 months of cART), and late chronic SIV/cART phase (7–12 months of cART).

**Figure 2:**
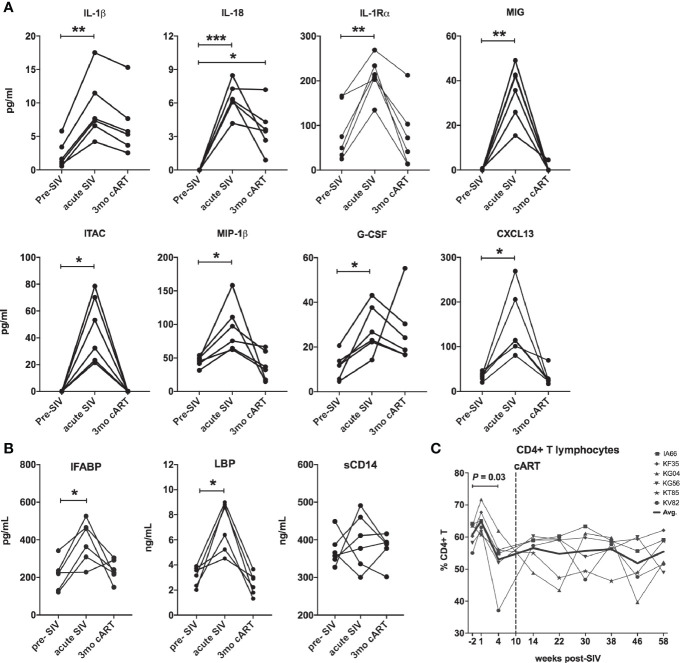
Downmodulation of acute phase pro-inflammatory cytokines and IEBD/MT biomarkers following cART initiation. **(A)** Longitudinal plasma levels of proinflammatory cytokines measured by Luminex assay in the 6 SIVmac251infected rhesus macaques. Data shown for cytokines/chemokines that were significantly different from baseline using one-way ANOVA with Dunnett’s multiple comparisons test. **(B)** Plasma levels of IFABP, a marker of enterocyte loss and generalized damage to the intestinal epithelium; LBP and sCD14, markers for host response to MT at pre-SIV, acute SIV (1-month post-SIV), and 3 months of cART. Two technical replicates were used for each time-point. Asterisks in all panels indicate significant differences between time points (*p < 0.05; **p < 0.01; ***p < 0.001). **(C)** Longitudinal percentage of CD4 T cells in PBMC through the course of SIV infection and cART treatment, showing a significant decline at 4 weeks post-SIV infection.

In agreement with previous work (26–29), acute SIV infection resulted in a surge in plasma cytokines, compared to baseline levels (**Figure 2**). Of the 40 analytes measured, all animals displayed significantly elevated levels of pro-inflammatory cytokines IL-1β, IL-18, IL-1Rα, MIG, ITAC, MIP-1β, G-CSF, and CXCL13 (**Figure 2A**). Further, in accordance with several studies demonstrating compromised gastrointestinal barrier integrity early during HIV/SIV infections (30–32), the study animals displayed a significant increase in the surrogate markers of IEBD and MT IFABP, and LBP (but not sCD14) at 1 month post-SIV infection (**Figure 2B**). This coincided with a significant decline in peripheral CD4 T lymphocytes frequencies (**Figure 2C**). After an initial increase in overall T lymphocyte numbers in the 1st week of infection followed by decreased CD4 T lymphocytes at 1-month post-SIV infection, the T lymphocytes resolved to baseline by the 1-month time-point, which was maintained it throughout set-point viremia and treatment phase (**Supplementary Figure 1**). Effective viral suppression with cART resolved the plasma inflammatory cytokines/chemokines and leaky gut biomarkers by 3 months of cART (**Figures 2A, B**).

### Recurrence of Systemic Inflammation and IEBD Biomarkers During the Chronic Phase of cART-Suppressed SIV Infection

**Figure 3:**
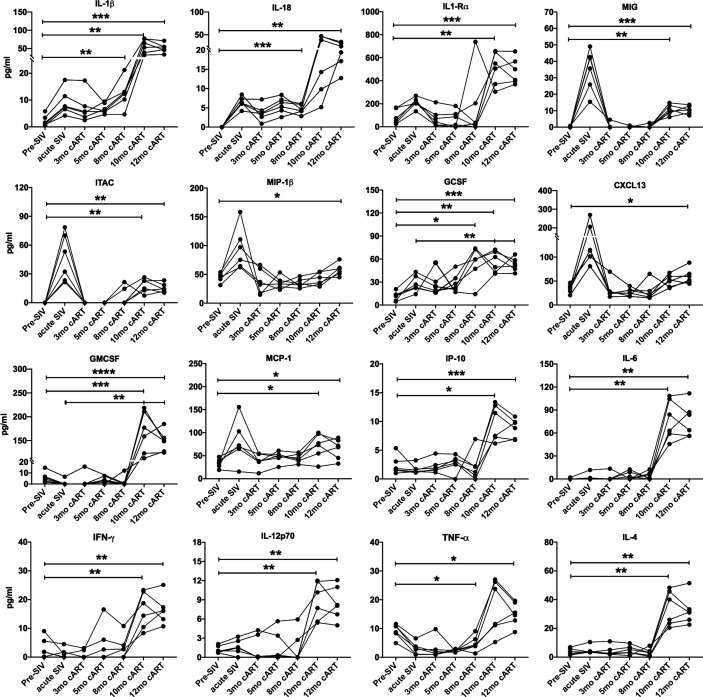
Increase in inflammatory cytokines during long-term cART. Longitudinal plasma levels of inflammatory cytokines measured by Luminex assay through the course of cART-suppressed SIV infection in the 6 SIVmac251infected rhesus macaques. One-way ANOVA with Tukey’s multiple comparisons test was used to determine significant differences between baseline and different time points post-SIV infection and cART. Asterisks in all panels indicate significant differences between time points (*p < 0.05; **p < 0.01; ***p < 0.001; ****p < 0.0001).

Despite continued treatment with cART (**Figure 1B**) multiple plasma inflammatory and IEBD markers re-emerged around 8–10 months of cART in all six macaques (**Figure 3**), suggesting the development of an inflammatory phenotype during chronic SIV infection that is independent of suppression of viremia and recovery of circulating CD4 T cell frequencies to baseline levels. Interestingly, at 10–12 months of cART, plasma levels of IL-1β (p = 0.007 for acute vs 10 month; p=0.001 for 12 month), GCSF (p=0.003 for acute vs 10 month; p = 0.008 for 12 month), IL-1Rα (p=0.04 for acute vs 10 month; p=0.013 for 12 month), and IL-18 (p = 0.03 for acute vs 12 month) were observed at higher levels than in the acute, pre-treatment phase (**Figure 3**). Notably, additional inflammatory cytokines including GM-CSF, MCP-1, IP-10, IL-6, IFN-γ, IL-12, and TNF-α were elevated only during the chronic phase following 8 months of cART (**Figure 3**), indicating that the plasma inflammatory cytokine signature during long-term controlled SIV infection is distinct from the cytokine storm of acute SIV infection. The Th2 cytokine, IL-4, was also significantly elevated at the 10- and 12-month time-point.

**Figure 4:**
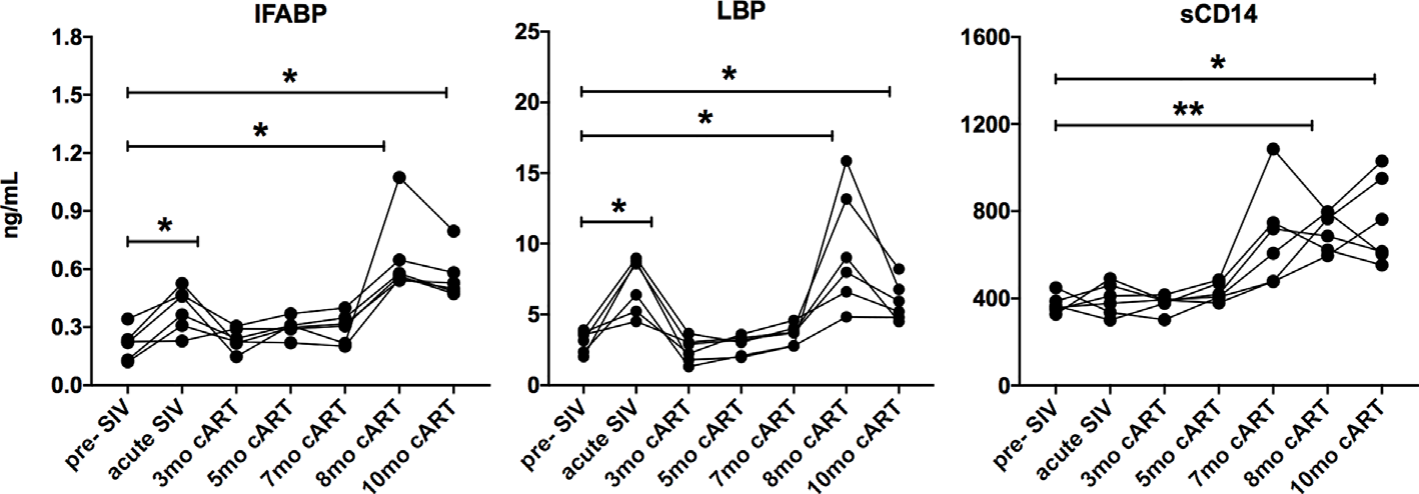
Increase in IEBD/MT biomarkers during long-term cART. Longitudinal plasma levels of IFABP, LBP, and sCD14 measured by ELISA through the course of cART-suppressed SIV infection the 6 SIVmac251infected rhesus macaques showing transient resolution following cART and increase around 8-10 months of cART. One-way ANOVA with Dunnett’s multiple comparisons test was used to determine significant differences from baseline. Asterisks in all panels indicate significant differences between time points (*p < 0.05; **p < 0.01).

Since the inflammatory mediators tracked with markers of IEBD and MT during acute SIV infection before treatment, plasma levels of IFABP, LBP, and sCD14 were also assessed longitudinally in parallel with cytokines/chemokines. Increased levels of IFABP and LBP were observed at 8–11 months post-cART (**Figure 4**). Further, consistent with several previous studies (33–35), we observed a surge in plasma sCD14 levels from 7 month onwards that were significantly higher than baseline (p=0.0018 at 8 month; p=0.03 at 11 month cART) as well as acute SIV/short-term antiretroviral treatment levels (p=0.003 at 8 month; p=0.047 at 11 month cART). The absence of any opportunistic infections or significant fluctuation in body temperature and weight of the animals (**Supplementary Figure 5**) suggested that the increase in plasma inflammatory markers was likely due to intrinsic changes in the host immune cell functions.

### Similarities Between the Plasma Inflammatory and Leaky Gut Phenotype of Chronic Treated SIV Infection and the Inflammaging Phenotype of Older SIV-Naïve Macaques

**Figure 5:**
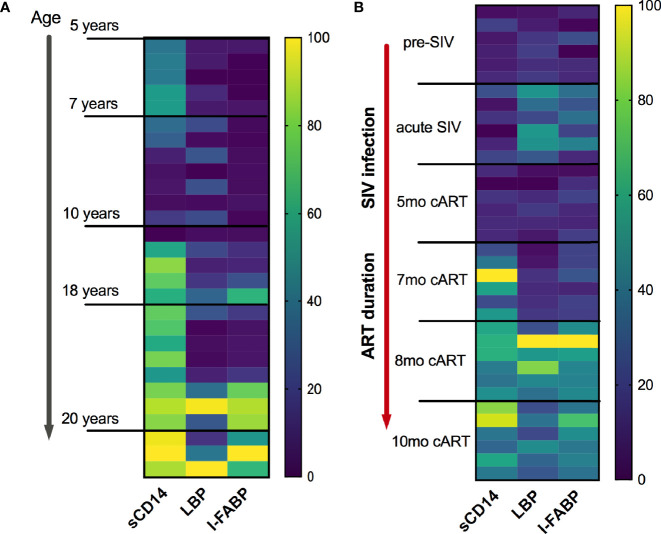
Similarities of plasma IEBD/MT biomarker profile between SIV-naïve inflammaging macaques and young SIV-infected macaques with long-term viral suppression. **(A)** Heat map showing the plasma concentrations of IFABP, LBP, and sCD14 in individual SIV-negative animals ordered from youngest to oldest. The heat map was normalized to the range for each individual marker so that the fold change represented by each color is consistent across markers. Blue = lowest value, Yellow = highest value for each marker. **(B)** Heat map showing the plasma concentrations of IFABP, LBP, and sCD14 in the 6 SIVmac251infected rhesus macaques at baseline (2 weeks pre-SIV infection), acute 1-month post-SIV, and through the course of cART till 10 months of treatment.

We recently reported that similar to humans (8), inflammaging phenotype in older SIV-naïve macaques is associated with a signature of increased circulating inflammatory cytokines and elevations in biomarkers of leaky-gut including IFABP, LBP, and sCD14 (13). Further analysis in this cross-sectional data to visualize the pattern of IFABP, LBP, and sCD14 expression (represented as a heat map for each individual across a row) revealed a gradient towards higher levels with increased age (**Figure 5A**; vertical columns) that was more pronounced from age 17 years and up. Likewise, in this longitudinal study of SIV-infected, cART suppressed young macaques, a strikingly similar pattern of increase in IFABP, LBP, and sCD14 developed during the chronic phase of infection (**Figure 5B**; vertical columns of the heat map). It should be noted that since the older SIV-naïve macaques are from a cross-sectional study, any contribution of differences in diet or social environment cannot be fully accounted for in this comparison. Nonetheless, it is noteworthy that IFABP and LBP that were maintained at baseline levels from 3–7 months of cART, increased significantly around 8 months and stayed elevated till 1 year of cART (**Figures 4** and **5B**). The increase in sCD14 was earlier and did not always track with IFABP and LBP within individuals in both the cross-sectional data and the longitudinal cohort (**Figures 5A, B**). Moreover, there was no significant correlation between sCD14 and the IEBD/MT markers IFABP and LBP (**Supplementary Figure 2A**), suggesting that factors besides MT may contribute to the increased secretion of sCD14, and that it likely is a more sensitive biomarker of inflammation. However, as anticipated, there was a highly significant correlation between IFABP and LBP (**Supplementary Figure 2B**) associated with the increase in inflammaging phenotype during the chronic phase of treated SIV infection.

### Reduced Frequencies of Peripheral Blood and Gut Mucosal Gammadelta (γδ) T Cells During Chronic cART Suppressed SIV Infection

**Figure 6:**
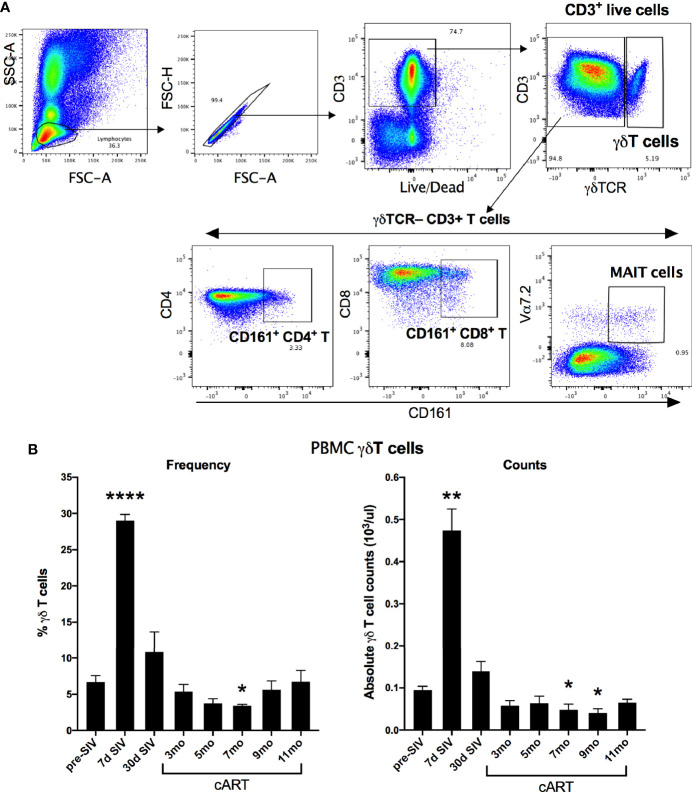
Longitudinal changes in circulating γδT cell frequencies and absolute counts in SIV-infected macaques during acute SIV infection and long-term viral suppression. **(A)** Representative gating schematic for γδT cells (CD3+ γδTCR+), Th17 cells (CD161+ CD4 T), Tc17 cells (CD161+ CD8 T), and MAIT cells (Vα24+ CD161+) in PBMC. **(B)** Frequencies of γδT cells in peripheral blood of study animals at baseline (2 weeks pre-SIV infection), acute SIV infection (7d and 1-month post-SIV), and through the course of cART till 11 months of treatment in the left panel; absolute numbers of γδT cells/μL of blood assessed by flow cytometry and complete blood cell counts (CBC) of lymphocytes at the indicated time points in the right panel. Bars represent mean ± SEM values and demonstrate a significant expansion of γδT cells at 7d post-SIV and a significant decline at 7-month cART time point. One-way ANOVA with Dunnett’s multiple comparisons test was used to determine significant differences between baseline and different time points post-SIV infection and cART. Asterisks indicate significant differences between time points (*p < 0.05; **p < 0.01; ****p < 0.0001).

**Figure 7:**
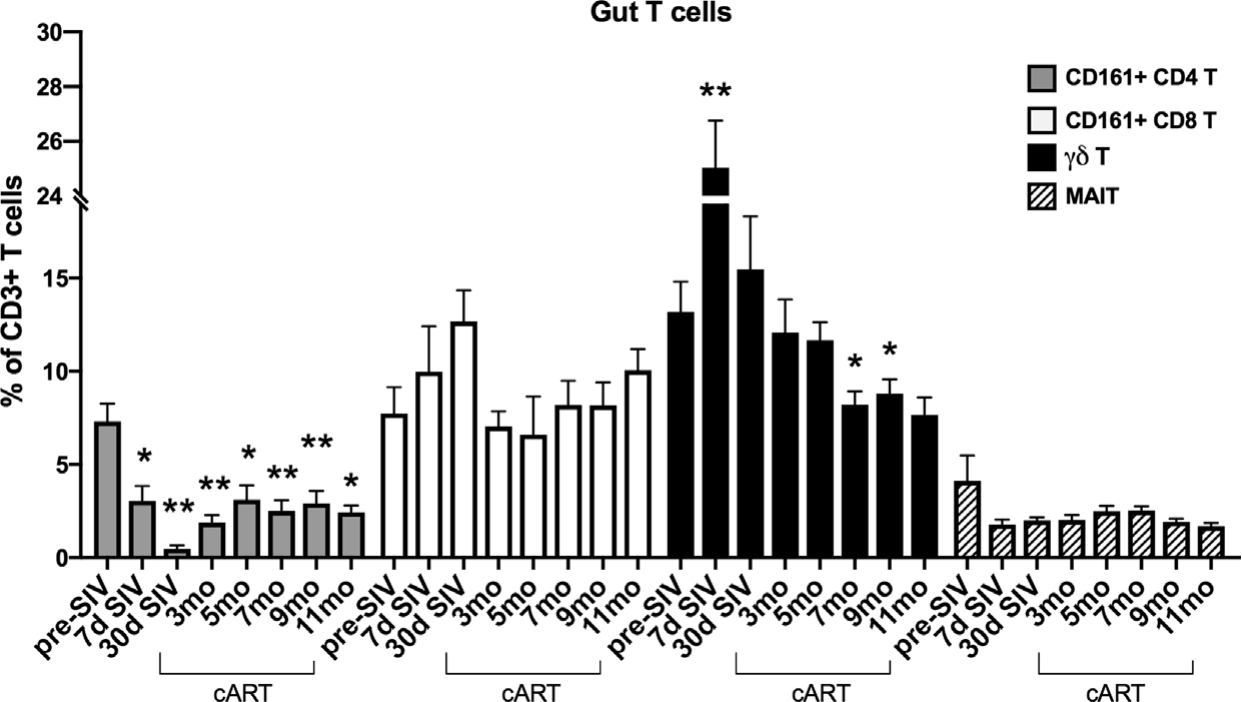
Longitudinal changes in frequencies of Th17 and innate IL-17 producing T cells in rectal mucosa of SIV-infected macaques during long-term viral suppression with cART. The fraction of Th17 and innate T cell subsets in lamina propria lymphocytes from rectal biopsies was assessed by flow cytometry, gated on live, singlet, CD3+ lymphocytes. Bar chart derived from flow cytometry data showing longitudinal changes in frequencies of diverse subpopulations of IL-17 producing T cells including Th17 cells (CD161+ CD4 T; grey bars), Tc17 cells (CD161+ CD8 T; open bars), γδT cells; black bars, and MAIT cells (Vα24+ CD161+; hatched bars) in the study animals at baseline (2 weeks pre-SIV infection), acute SIV infection (7d and 1-month post-SIV), and through the course of cART till 11 months of treatment. Bars represent mean ± SEM values and demonstrate a significant and persistent decline in classical CD4+ Th17 cells and an early increase in γδT cell frequencies following SIV infection. One-way ANOVA with Dunnett’s multiple comparisons test was used to determine significant differences between baseline frequency and different time points post-SIV infection and cART. Asterisks in all panels indicate significant differences between time points (*p < 0.05; **p < 0.01, ***p < 0.001).

The significant increase in circulating biomarkers of IEBD/MT and plasma inflammatory mediators in the chronic phase of cART-suppressed SIV infection in our study animals demonstrated an association of impaired epithelial barrier function with systemic inflammation. Given the role of IL-17/IL-22-producing lymphocytes in contributing to the maintenance of intestinal epithelial barrier integrity (36), we investigated the relationship between IL-17/IL-22 cytokine effector functions of classical and innate Th17-type cells in blood and gut mucosa and levels of IEBD/MT and plasma inflammatory biomarkers. We and others have demonstrated that besides the classical CD161+ CD4+ Th17 cells, subsets of T cells including CD161+ CD8 T cells (Tc17), gammadelta (γδ) T cells, and CD161+Vα7.2+ MAIT cells are capable of IL-17/IL-22 effector functions and are particularly enriched in the primate intestinal mucosa (23, 37–39). During early acute SIV infection in our study, peripheral blood γδ T cells displayed a very significant increase in frequency (p<0.0001) and absolute numbers (p=0.003) that returned to baseline by 1 month post-infection (**Figure 6**). However, later during chronic phase of infection, γδ T cell numbers declined to significantly low levels at the 7–9-month cART time-points (p=0.046 and 0.01 respectively; **Figure 6**). In agreement with previous studies, a persistent significant decline in Th17 cell frequencies was observed in the rectal mucosa through the course of SIV infection and suppressive cART in this study (**Figure 7**). However, no significant differences were observed in the frequencies of mucosal Tc17 cells and MAIT cells through the course of chronic treated SIV infection (**Figure 7**). Interestingly, gut mucosal γδ T cell frequencies increased significantly by 7 days post-SIV infection (p=0.005) and returned to baseline during early cART (**Figure 7**), suggesting an early response to ongoing viral replication in the gut. However, during the long-term treatment phase, a decline in gut mucosal γδ T cells was observed reaching significance at the 7–9-month cART time-points (p=0.04 and 0.03 respectively; **Figure 7**) demonstrating similar kinetics of responses between blood and gut mucosal compartments. Notably, this decline in gut mucosal γδ T cells preceded the elevation in plasma IFABP, LBP and sCD14 (**Figure 4**), suggesting the loss of γδ T cells may play a role in the subsequent increase of leaky gut biomarkers during chronic treated SIV infection. Thus, among the various subsets of Th17-type T cells in the gut mucosa, significant changes in γδ T cell frequencies aligned with subsequent increase in plasma inflammatory and leaky-gut markers during long-term cART suppressed SIV infection (**Figures 3** and **4**).

### Impaired Gut Mucosal γδ T Cell Effector Function Is Associated With Development of IEBD and Systemic Inflammation During Long-Term cART Suppressed SIV Infection

**Figure 8:**
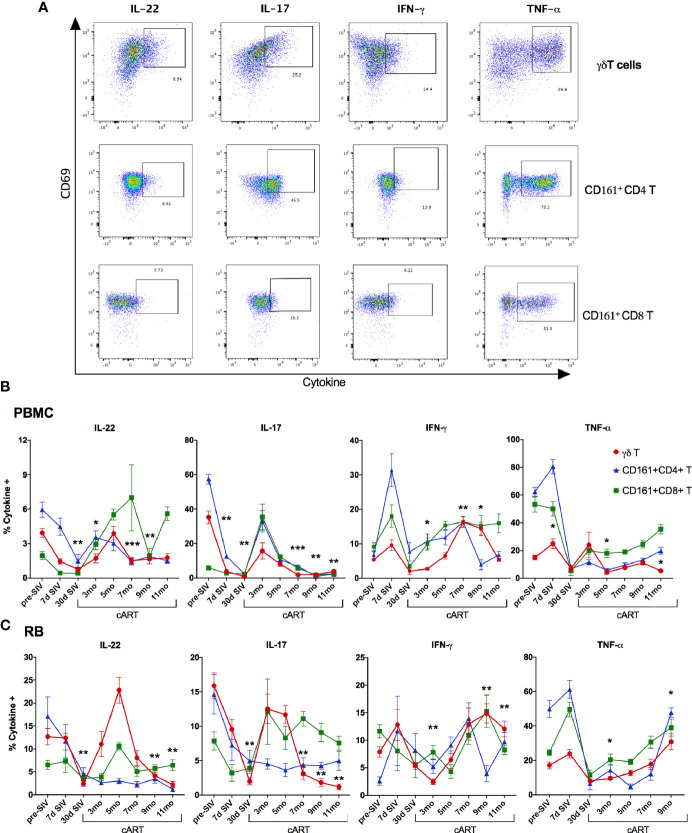
Recovery of IL-17/IL-22 -producing γδT cells during short-term cART in blood and rectal mucosa followed by significant decline during long-term cART-suppressed SIV infection. **(A)** Representative flow cytometry plots showing intracellular cytokine staining of γδT cells, CD161+ CD4 T cells, and CD161+ CD8 T cells in PMA/Ionomycin stimulated PBMC. Flow cytometry analysis demonstrated a decrease of intracellular IL-17- and IL-22-production in Th17 cells (CD161+ CD4 T; blue symbol/line), Tc17 cells (CD161+ CD8 T; green symbol/line), and γδT cells (red symbol/line) during acute SIV infection prior to cART in PBMC **(B)**, and in rectal mucosa **(C)**, which returned to baseline by 3-month cART in both Tc17 and γδT cells but not in Th17 cells. Significant reduction in IL-17- and IL-22-production of γδT cells was observed again at 7-month cART time point along with increase in IFN-γ production. One-way ANOVA with Dunnett’s multiple comparisons test was used to determine significant differences between baseline and different time points post-SIV infection and cART. Asterisks indicate significant differences in γδT cell cytokine production between time points (*p < 0.05; **p < 0.01, ***p < 0.001).

**Figure 9:**
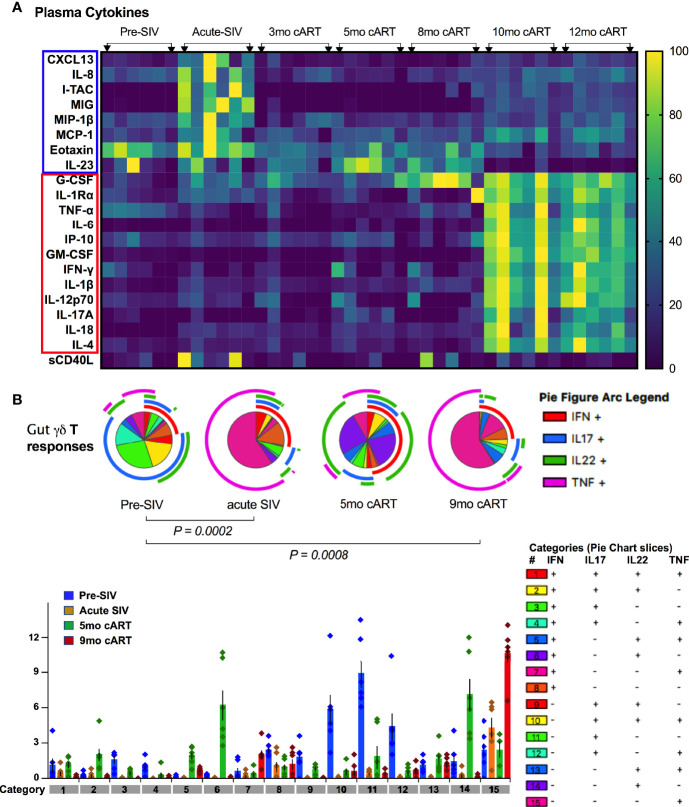
Plasma inflammatory markers align with significant loss of polyfunctional IL-17/IL-22 cytokine responses in gut mucosal γδT cells. **(A)** Kinetics of plasma cytokine/chemokine levels during long-term cART-suppressed SIV infection. Heat map comparing plasma analytes at baseline (2 weeks pre-SIV infection), acute SIV infection (1-month post-SIV), and through the course of cART till 12 months of treatment. Rows represent each analyte and columns represent each animal at indicated time points. Average cytokine concentration of two technical replicates in pg/ml was normalized to the range for each individual marker so that the fold change represented by each color is consistent across analytes. Blue = lowest value, Yellow = highest value for each analyte). Cytokines/chemokines upregulated in acute SIV infection are highlighted in blue box and those upregulated during long-term cART (10-12 months) are in red box, showing a distinct profile of plasma inflammatory mediators during chronic treated SIV infection. **(B)** Skewing of dominant IL-17/IL-22 polyfunctional responses towards a dominant Th1-type response during acute SIV infection pre-treatment with cART and later during long-term cART. Pie charts (top) and bar chart (bottom) comparing changes in polyfunctional γδT cell responses from baseline, pre-SIV infection through acute SIV infection and during short-term (5-month) and long-term (9-month) cART in the six SIV-infected macaques. The pie charts represent the average frequencies of active cytokine-producing cells making every possible combination of IL-17, IL-22, TNF-α and IFN-**γ**. The segments within the pie chart denote populations producing different combinations of cytokines and are color coded (as shown in the legend for categories of every combination of cytokines). The arcs around the circumference indicate the particular cytokine produced by the proportion of cells that lie under the arc. Parts of the pie surrounded by multiple arcs represent polyfunctional cells. The pie arc legend shows IFN-**γ** in red, IL-17 in blue, IL-22 in green, and TNF-α in purple. p values were computed using the SPICE permutation test (25).

We next investigated the γδ T cell effector functions and activation status in the context of acute SIV-infection, short-term cART, and during the chronic phase of treated SIV infection (**Figure 8** and **Supplementary Figure 6**). The rectal mucosal γδ T cells in healthy SIV-naive macaques displayed a dominant IL-17 and IL-22 cytokine response to PMA/Ca Ionomycin stimulation that is comparable to the classical Th17 subsets (**Figures 8** and **9B**). However, production of the Th1 cytokine, TNF-α, was lower in γδ T cells in comparison to both Th17 and Tc17 cells. We have previously shown that CD161+ Th17 type mucosal T cells, besides being capable of both Th17 and Th1 cytokine production, were more polyfunctional than CD161-negative T cells (23). However, it remains unclear whether there are any differences in the polyfunctionality or the balance between Th1 and Th17 cytokine responses between the subsets of gut mucosal Th17 type T cells. Thus, we compared the cytokine response of rectal mucosal γδ T cells with Th17 and Tc17 cells for every combination of the four cytokines IL-17, IL-22, TNF-α, and IFN-γ. The results demonstrate that overall cytokine responses of rectal mucosal γδ T cells are significantly different from that of classical Th17 cells (p=0.0003) and Tc17 cells (p=0.0008), with significant greater frequencies producing various combinations of IL-17 and IL-22 (**Supplementary Figure 3**). This suggests that under steady-state conditions, gut mucosal γδ T cells have substantially enhanced IL-17/IL-22 polyfunctionality among other subsets of Th17-type cells.

As widely reported in HIV/SIV infection (40–43), we observed a significant decline in IL-17 and IL-22 cytokine producing ability of Th17 cells within a month of SIV infection. Although there was a partial recovery of Th17 cytokine functions in peripheral blood during early cART (3 months of treatment, **Figure 8A**), the rectal mucosal Th17 cells displayed sustained loss in IL-17/IL-22 production throughout the course of cART (**Figure 8B**). Indeed, a persistent impact of SIV infection was observed on both frequencies (**Figure 6**) and epithelial barrier-protective effector functions of mucosal Th17 cells. Likewise, IL-17 and IL-22 production was significantly reduced in γδ T cells (**Figure 8**), which was evident earlier in PBMC (7 days post-SIV; **Figure 8A**) than in the rectal mucosa (30 days post-SIV; **Figure 8B**). There was an early increase in IFN-γ and TNF-α production that returned to baseline by 30 days post-SIV in blood and rectal mucosal γδ T cells (**Figures 8A, B**). The kinetics of the Th1/Th17 cytokine response was similar between γδ T and Th17 cells in blood and rectal mucosa during acute SIV infection prior to cART (**Figure 8**). However, unlike the continued decline in IL-17/IL-22 cytokine responses of rectal mucosal Th17 cells, γδ T and Tc17 cells demonstrated a recovery to baseline following cART initiation (**Figure 8B**). Intriguingly, even though IL-17/IL-22 effector functions were maintained during short-term (3–5 months) cART, a significant loss of IL-17 and IL-22 producing ability was observed in blood and rectal mucosal γδ T cells around 7 month of cART (**Figure 8**). Notably, during this stage of treated chronic SIV infection, γδ T cells displayed significantly higher production of IFN-γ in both peripheral blood (p=0.003 at 7 months, p=0.014 at 9 months cART) and rectal mucosa (p<0.01). Additionally, a trend towards enhanced TNF-α production was also observed in rectal mucosal γδ T cells that reached significance (p=0.016) at 9 months post-cART (**Figure 8**). Thus, despite return to baseline during early cART and effective suppression of viremia, γδ T cells exhibited significant changes in effector functions reflecting a specific loss of Th17 type cytokine producing ability and skewing towards Th1 type cytokine responses. This dysfunction aligned with the reduced frequencies of rectal mucosal γδ T cells, thus, strongly suggesting a role in the subsequent increase in plasma IEBD/MT biomarkers and inflammation observed in this study (heat maps in **Figures 5B** and **9A**). Indeed, comparison of gut mucosal γδ T cell polyfunctional responses for IL-17, IL-22, TNF-α and IFN-γ through the course of SIV infection and cART demonstrated an initial loss of Th17 cytokine functions during acute SIV infection followed by return to baseline by 5 months of cART and subsequently a significant decline in IL-17/IL-22 polyfunctional, and increased TNF-α and IFN-γ mono-functional responses (pie charts in **Figure 9B**). In contrast there was a sustained loss of polyfunctional cytokine responses in CD4 Th17 cells throughout acute and treated SIV infection (**Supplementary Figure 4A**) and no significant change in Tc17 cells until 9 months of cART (**Supplementary Figure 4B**).

### Perturbation in Vδ1 and Vδ2 Subset Ratios During Chronic Treatment Correlates With Loss of Mucosal Th17-Type Cytokine Functions and Increased Plasma IEBD/MT Biomarkers

**Figure 10:**
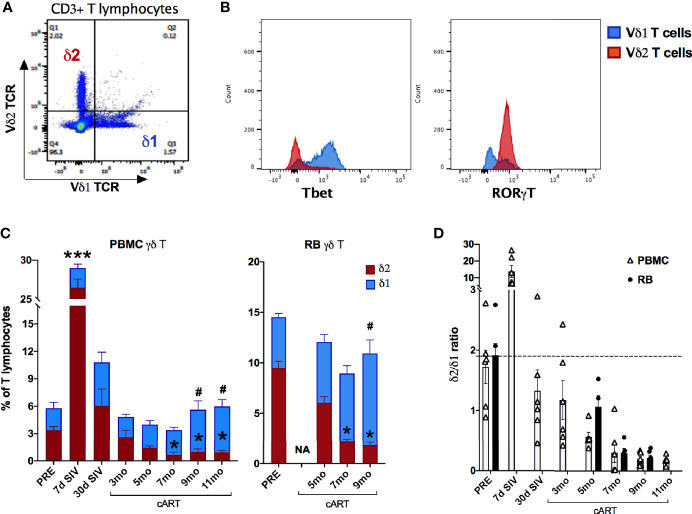
Significant changes in blood and rectal mucosal γδT cell ratios due to decline in Vδ2 and increase in Vδ1 cells during long-term cART. **(A)** Representative flow cytometry plot showing Vδ1 and Vδ2 subset staining on CD3+ T lymphocytes. **(B)** Representative histogram plot showing expression of the transcription factors Tbet and RORγT on Vδ1 and Vδ2 subsets. **(C)** Relative frequencies of Vδ1 and Vδ2 γδT cell subsets as a fraction of total T lymphocytes from PBMC and rectal biopsies (RB) at indicated time points measured by flow cytometry. Stacked bar graphs showing significant differences from pre-SIV time point using One-way ANOVA with Dunnett’s test and represented by * for Vδ2 subset in red bars and # for Vδ1 subset in blue bars. **(D)** Bar graph comparing Vδ1/Vδ2 ratios between PBMC (open bars) and RB (black bars) through the course of SIV infection and cART. Missing bars at some time points for RB are due to poor cell recovery at those time points. The dashed line represents average Vδ1/Vδ2 ratios of two baseline time points.

Primate γδ T cells have a relatively restricted repertoire of V gene segments, with Vδ1 and Vδ2 being the most commonly used Vδ gene segments in blood and mucosal tissues (44–46) and are termed Vδ1 and Vδ2 subsets. An inversion of the typical Vδ2/Vδ1 subset ratio in circulating blood has been associated with HIV/SIV infection (16, 45, 47), however the impact of chronic treated infection on gut mucosal Vδ1 and Vδ2 subsets remains unclear. Since the overall γδ T cell compartment displayed significant perturbations in frequencies and function aligning with the recurrence of IEBD and inflammation during the chronic phase of treated SIV infection in our study, Vδ1 and Vδ2 subset composition of blood and rectal mucosal γδ T cells were examined over the course of long-term viral suppression with cART. The average Vδ2/Vδ1 ratio in blood and rectal mucosa was 1.7-1.9 at baseline prior to SIV challenge (**Figure 10B**). Notably, by 7-day post-SIV infection there was a remarkable increase in circulating Vδ2 frequencies, resulting in significantly higher numbers of total γδ T cells (**Figure 7**) and higher Vδ2/Vδ1 ratio (average of 13.76; **Figure 10B**) that returned to just below baseline during early cART.

**Figure 11:**
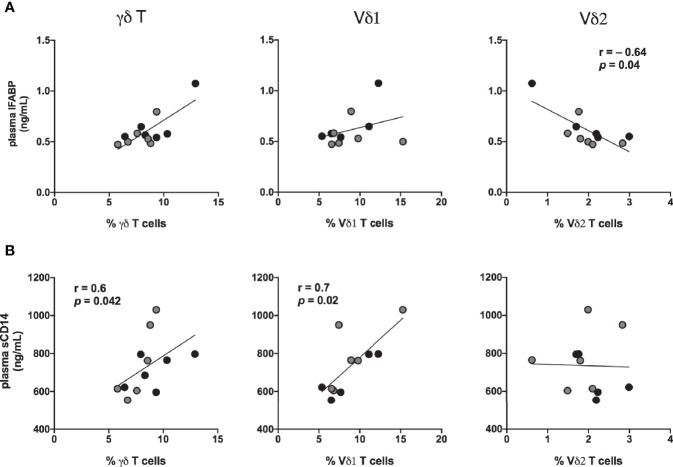
Association of IFABP and sCD14 levels with inversion of rectal mucosal γδT cell subset ratios during chronic treated SIV infection. Spearman Rank correlation between frequencies of total γδT cells and the specific Vδ1 and Vδ2 in rectal mucosa and: **(A)** plasma concentration of IFABP, and **(B)** plasma sCD14 levels at 7-8 months (black circles) and 9–11 months (grey circles) of cART in the study animals. Statistical correlations were investigated by the Spearman correlation coefficient and 95% confidence limits. Statistical significance (*p* values) and Spearman’s coefficient of rank correlation (r) are shown for negative correlation of rectal Vδ2 frequencies with plasma IFABP and positive correlation of Vδ1 frequency with sCD14 levels.

The Vδ1 and Vδ2 subset distribution in rectal mucosal lymphocytes could only be evaluated at baseline and chronic SIV infected time-points owing to the low cell yields during acute SIV infection and early cART time-points. For the time-points analyzed, the dynamics of Vδ2/Vδ1 ratio in rectal mucosa resembled the dynamics observed in blood (**Figures 10A, B**). Significantly reduced Vδ2 frequencies from 7-months cART onwards with a concomitant increase in Vδ1 frequencies was observed in PBMC and rectal biopsies (**Figure 10A**) resulting in significant decline of the Vδ2/Vδ1 ratio from baseline (**Figure 10B**). Furthermore, during the chronic phase of treatment, the specific loss of rectal mucosal Vδ2 subsets significantly correlated with plasma IFABP levels (**Figure 11A**). On the other hand, elevated levels of mucosal Vδ1 cells displayed significantly correlated with plasma sCD14 concentrations (**Figure 11B**). Taken together, the overall decline in IL-17/IL-22 functions along with significant loss of Vδ2 cells, particularly in the rectal mucosa, coupled with significant correlations with plasma IFABP levels, points towards a likely role for Vδ2 cell impairment in the loss of gut barrier functions, despite long-term effective cART in SIV-infected macaques.

## Discussion

Gut barrier breakdown and persistent inflammation are hallmarks of chronic HIV infection despite long-term treatment with antiretroviral drugs, and PLWH have an immune aging/inflammaging phenotype. γδT cells are a subset of innate Th17-type T cells that are key players in gut barrier function and immune activation (48), yet their role in inflammaging during the course of treated HIV infection is not understood. To investigate the role of γδT cell functions in the onset of inflammaging phenotype during cART suppressed SIV infection, this longitudinal study comprehensively examined the kinetics of plasma inflammatory and IEBD/MT markers through the course of treated SIV infection and evaluated γδT cell frequencies and cytokine effector functions in blood and gut mucosa. We provide evidence that, (i) the recurrence of inflammation during the chronic phase of effectively suppressed SIV infection has a distinct signature from that observed during the acute viremia phase, (ii) the increase in IEBD and inflammatory markers of chronic treated SIV infection resembles the inflammaging phenotype of SIV-naïve older macaques, and (iii) impaired γδT cell IL-17/IL-22 effector functions and dysregulated Vδ1 and Vδ2 subset ratios in both peripheral blood and rectal mucosa precede the development of inflammaging phenotype during long-term treated SIV infection. Thus, utilizing the macaque model of mucosal HIV infection and successful viral suppression with cART, this study demonstrates that γδT cell dysfunction may contribute to the persistent inflammation of aviremic individuals living with HIV.

Several studies have examined inflammaging phenotype in PLWH by evaluating large datasets comprised of wide age-ranges, often grouped as young and old categories (8, 49–51). Since biological aging is a continuum and is impacted by several factors including HIV infection itself, elucidating the immune mechanisms during virus infection and its treatment before the onset of biological aging is an important first step toward understanding the process of inflammaging in PLWH. Aging rhesus macaques typically begin exhibiting relatively higher levels of circulating cytokines/chemokines associated with inflammaging around 18 years of age (13, 52). In this study, we evaluated the development of inflammaging phenotype through the course of treated SIV infection in young adult macaques (5–10 years old) well before the onset of non-infectious inflammaging. As reported in early HIV infection (11, 53, 54), we observed that systemic inflammation following acute mucosal (IR) SIV infection was associated with elevation in plasma LBP and IFABP, markers of MT and enterocyte damage respectively. It is important to note that during acute SIV infection prior to treatment with cART, while we observed a correlation between IFABP and LBP, we did not establish any relationship between either IFABP, or LBP and sCD14. Indeed, there was no significant increase in plasma levels of sCD14 during the inflammatory response of acute SIV infection despite a set-point viremia of 5.8 log and increase in several pro-inflammatory mediators including IL-1β, IL-18, IL-1Rα, MIG, ITAC, MIP-1β, G-CSF, and CXCL13 as well as elevated IFABP and LBP levels. Despite its wide use as a plasma MT marker, sCD14 is not an exclusive marker of ongoing translocation of bacterial by-products from gut lumen into blood circulation but also serves as a biomarker of monocyte activation (55) and there are conflicting reports on the levels of plasma sCD14 reflecting MT in PLWH (55, 56) and SIV-infected macaques (31, 57). It is likely that a more significant monocyte/macrophage activation and prolonged intestinal mucosal damage may be required for significantly elevated plasma sCD14 levels. Thus, the increase of sCD14 in the chronic phase of cART (7 months onwards in this study) possibly reflects a combination of persistent monocyte/macrophage activation and MT beyond the levels observed in the acute phase of SIV infection.

To understand the potential immune mechanisms of IEBD and MT associated with persistent inflammation during chronic cART-suppressed SIV infection, we longitudinally compared diverse subpopulations of Th17-type immune cells in blood and rectal mucosa. The main finding was the detection of a significant correlation of IEBD and systemic inflammation with a specific loss of Th17-type γδT effector functions during long-term suppressive ART. γδT cells represent a unique subset of effector T cells that can traffic to tissues, are enriched at gut mucosa where HIV prevails, and selectively target cancer or virally infected cells (47). Chronic HIV/SIV infection is associated with impaired proliferative responses of γδT cells (58, 59) and inversion of Vδ2:Vδ1 subset ratios (16, 45, 60). However, the role of gut mucosal γδT cell functions in IEBD, MT and persistent inflammation during the course of treated HIV infection remains unclear. In this prospective study, we demonstrated that gut mucosal γδT cells are a dominant source of polyfunctional IL-17 and IL-22 responses among the diverse subsets of Th17-type immune cells including classical Th17, Tc17, and MAIT cells in healthy SIV-naïve macaques. Furthermore, gut mucosal γδT cells displayed important differences in the kinetics of IL-17/IL-22 cytokine responses from other Th17-type cells through the course of treated SIV infection. Notably, an early expansion of γδT cells in all of the study animals within a week of SIV infection, particularly in rectal mucosa, suggests a likely role in compensatory immune mechanisms for the preferential and sustained loss of gut CD4+ Th17 cells. However, this early expansion was not sufficient to prevent subsequent IEBD and MT, as plasma IFABP and LBP increased later in acute SIV infection, prior to treatment with cART. Indeed, despite the transient expansion in numbers, both peripheral and rectal γδT cells displayed a significant decline in IL-17 and IL-22 cytokine responses indicating that a specific decline in γδT IL-17/IL-22 effector functions, besides the significant gut mucosal Th17 depletion, likely contributed to the overall loss of epithelial barrier integrity during acute phase of infection. It is likely that the innate immune activation and the cytokine storm induced by acute infection modulated γδT cell functions toward loss of Th17-type cytokine expression and increases in Th1 cytokine production. This is supported by the increased production of TNF-α and IFN-γ by both blood and rectal γδT cells observed in this study.

Intriguingly, in contrast to the widely reported expansion of circulating Vδ1 cells during chronic HIV/SIV infection (14, 21), we found that a significant increase in Vδ2 rather than Vδ1 subset contributed to the early expansion of γδT cell numbers within a week of SIV infection. However, our results are consistent with an earlier report in rhesus macaques (61) showing peripheral expansion of Vδ2 cells that returned to baseline by 1-month post-SIV infection. Thus, it is likely that a very early expansion of Vδ2 subset occurs in HIV infection as well, which needs further investigation. In the lack of data on Vδ1 and Vδ2 subset distribution in rectal mucosa during the acute pre-treatment phase of SIV infection in the current study, it remains to be confirmed whether a similar increase of Vδ2 cells occurs in the gut mucosal compartment. However, based on the comparable kinetics of the changes in subset ratios between blood and rectal mucosa at baseline and throughout the treatment phase, we speculate that a similar expansion of gut mucosal Vδ2 cells contributed to the increase in total γδT cell frequencies observed 1-week post-SIV infection in rectal mucosa. Normalization of this early switch in γδT subset ratios during suppression of viremia with cART and recovery of polyfunctional IL-17/IL-22 cytokine functions indicate that the perturbations in γδT cell functions and subset balance were driven by active viral replication prior to initiation of cART. Moreover, the significantly enhanced IL-22 responses in gut mucosal γδT cells during early viral suppression with cART suggests an active role in epithelial repair process of the IEBD caused during acute SIV infection. IL-22 is required for the preservation of the intestinal epithelial barrier and wound healing processes *via* the maintenance and proliferation of epithelial stem cells (62, 63). However, in agreement with other reports of treated HIV infection (47), the recovery of γδT effector functions and subset ratios was not maintained through long-term cART and it was reversed to significantly lower Vδ2 and higher Vδ1 subset frequencies around 7 months of continued treatment and effective viral suppression. Importantly, this inversion of Vδ1 and Vδ2 subset ratio along with the recurrent loss of IL-17- and IL-22-producing effector functions preceded the subsequent increase in plasma IFABP, LBP, and sCD14 levels. This suggests that IL-17- and IL-22-producing γδT cells may have an essential role in the maintenance of the gut epithelial barrier integrity during effective suppression of viremia with cART, and that loss of these cells during chronic treated SIV infection may contribute to increase in IEBD and MT biomarkers. The mechanisms of dysregulation in γδT cell numbers and function during long-term suppressed SIV infection are unclear at this point. Several factors including ongoing viral replication in the gut tissue and/or long-term exposure to cART drugs, changes in the gut microbiome (64), or depletion of helper CD4+ T cells (65), etc. are likely to drive γδT cell dysfunction, thereby contributing to IEBD and MT. Although our data are not conclusive to demonstrate that this link is causative, it is noteworthy that plasma IFABP levels showed a significant negative correlation with frequency of gut mucosal Vδ2 T cells during long-term cART phase. This argues for a causative role of decline in epithelial barrier-protective effector functions of Vδ2 cells and the emergence of a leaky gut phenotype. On the other hand, the attendant increase in Vδ1 subset and enhanced Th1 cytokine production may reflect a response of Vδ1 cells to leaky gut mediated systemic inflammation, as Vδ1 cells proliferate in response to inflammatory cytokines (66). Thus, besides the association of impaired Vδ2 functions with leaky gut biomarkers, increases in7nbsp;Vδ1 cells and their corresponding inflammatory cytokine production in the gut mucosa may further contribute to systemic inflammation during chronic cART-treated SIV infection.

Notably, the resurgence of plasma IEBD/MT biomarkers and inflammatory cytokine/chemokine markers resembled the inflammaging phenotype of older SIV-naïve macaques comprising of elevated levels of IFABP, LBP, sCD14, GM-CSF, IL-1β, IL-12, IL-6, and TNF-α (13), and was distinct from the cytokine storm of acute SIV infection phase. A similar inflammaging phenotype has been described in chronic HIV infected persons (8). Based on our data, we propose that the loss of Vδ2 T cells initiated a vicious cycle of inflammation with the inversion of Vδ1/Vδ2 subset ratio and dysregulated cytokine effector functions in the residual γδT cells thereby contributing to development of inflammaging phenotype. Of note, a recent study has demonstrated that among diverse lymphocyte subsets including NK cells, T cells, Tregs, and iNKT cells, only γδT cell phenotype (activated/exhausted TIGIT+PD-1+ phenotype associated with plasma pro-inflammatory profile) could distinguish inflammaging in aviremic HIV infected individuals from HIV-negative younger and older individuals (50), implicating γδT cells as the key inflammatory driver in cART-suppressed HIV infection. Moreover, HIV elite/viral controllers maintain significantly higher Vδ2 frequencies than untreated or cART-treated PLWH and display preserved IL-17 function and lower immune activation (67, 68). Further research is warranted in order to deeply understand the precise mechanism of HIV/SIV-mediated γδT cell dysfunction in gut mucosa and the specific role of Vδ1 and Vδ2 subsets in driving leaky gut-mediated inflammaging process. Our observation opens the possibility that augmenting IL-17/IL-22 cytokine functions of γδT cells may ameliorate persistent systemic inflammation of chronic treated SIV infection by promoting the maintenance of epithelial barrier functions and preventing MT. Clinical augmentation of Vδ2 cells has been demonstrated in cancer patients through administration of bisphosphonates, which are a class of compounds recognized only by the Vδ2 subset of γδT cells (69, 70). A strength of this study is the longitudinal assessment of the development of inflammation during the course of effectively suppressed chronic SIV infection, which enabled the assessment of changes in immune cell functions in blood and gut mucosa prior to the emergence of leaky gut phenotype and systemic inflammation associated with chronic treated lentiviral infection. However, a limitation of our study design is the use of only female rhesus macaques to enable direct comparison with the inflammaging phenotype established in aging female rhesus macaques in our previous study. It will be of interest to evaluate whether there are gender-based differences in the functions of γδT cells and their role in the development of inflammaging phenotype in male macaques with chronic SIV infection and long-term cART.

In summary, we demonstrated that the inflammatory cytokine/chemokine signature of long-term cART is distinct from acute SIV infection. Furthermore, we have shown that significant changes in gut mucosal γδT cell functions, particularly loss of Vδ2 cells and impaired IL-17/IL-22 producing ability are associated with development of systemic inflammation during chronic treated SIV infection in rhesus macaques. This study contributes to the current understanding of γδT cell dysregulation as a mechanism of HIV/SIV immune evasion and provides deeper insights into the potential role of γδT cell dysfunction in the re-emergence of leaky gut and inflammatory phenotype following initial control of immune activation during effective suppression of viremia by ART. Further studies aimed at identifying the mechanisms driving γδT cell dysfunction and increased gut permeability during aviremic SIV infection are required to test its potential as an immune-based intervention to improve mucosal homeostasis and reduce HIV-associated chronic inflammation.

## Data Availability Statement

The original contributions presented in the study are included in the article/**Supplementary Material**. Further inquiries can be directed to the corresponding author.

## Ethics Statement

The animal study was reviewed and approved by Tulane University Institutional Animal Care and Use Committee (IACUC).

## Author Contributions

NR conceived the project, designed experiments, and supervised the work. EW, NS, and GG performed experiments and analyzed data. BG is the veterinarian in this study. JM provided additional samples and PK, DW, RV, and SJ helped with overall data interpretation. All authors contributed to the article and approved the submitted version.

## Funding

This study is supported by the NIGMS, NIH award P20GM103629 awarded to SJ and NR, in part by the NIH grant P51OD011104 to Tulane National Primate Research Center, and in part by the Intramural Research Program, National Institute on Aging, NIH.

## Conflict of Interest

The authors declare that the research was conducted in the absence of any commercial or financial relationships that could be construed as a potential conflict of interest.

## Supplementary Material

Supplementary Figure 1 Longitudinal assessment of percent and absolute number of T cells during the course of SIV infection and cART. **(A)** Percent CD3+ T lymphocytes and CD8+ T lymphocytes in PBMC at pre-SIV (week -2) and following SIV infection up to 50 weeks. **(B)** Absolute counts of total CD3+, CD8+ and CD4+ T lymphocytes per μl of peripheral blood at the indicated time points.

Supplementary Figure 2 Highly significant association of IFABP with LBP levels during long-term cART suppressed SIV infection. Spearman Rank correlation between plasma concentration of LBP and IFABP during: **(A)** acute SIV infection and early cART (1-month post-SIV: blue circles, 2-month cART: green circles), and **(B)** chronic cART-treated SIV infection (7-month: black circles, 8-month: crimson circles, and 11-month: red circles) in the study animals. Statistical significance (*p* values) and Spearman’s coefficient of rank correlation (r) are shown. Correlations of sCD14 with LBP and IFABP during: **(C)** acute SIV infection, and **(d)** chronic treated SIV infection showing no significant associations.

Supplementary Figure 3 γδT cells are a dominant source of polyfunctional IL-177/IL-22 responses in the gut. Pie charts comparing polyfunctional Th1/Th17 cytokine responses in Th17 (CD161+CD4+ T), γδT and Tc17 (CD161+CD4 = 8+ T) cells under steady state conditions. The pie charts represent the average frequencies of active cytokine-producing cells making every possible combination of IL-17, IL-22, TNF-α and IFN-**γ**. The segments within the pie chart denote populations producing different combinations of cytokines and are color coded. The arcs around the circumference indicate the particular cytokine produced by the proportion of cells that lie under the arc. Parts of the pie surrounded by multiple arcs represent polyfunctional cells. The pie arc legend shows IFN-**γ** in red, IL-17 in blue, IL-22 in green, and TNF-α in purple. p values were computed using the SPICE permutation test (25).

Supplementary Figure 4 Changes in rectal mucosal Th17 and Tc17 polyfunctional responses during long-term SIV suppression with cART. Pie charts comparing polyfunctional Th1/Th17 cytokine responses in **(A)** Th17 (CD161+CD4+ T), and **(B)** Tc17 (CD161+CD4 = 8+ T) cells at indicated time points. The pie charts represent the average frequencies of active cytokine-producing cells making every possible combination of IL-17, IL-22, TNF-α and IFN-**γ**. The segments within the pie chart denote populations producing different combinations of cytokines and are color coded. The arcs around the circumference indicate the particular cytokine produced by the proportion of cells that lie under the arc. Parts of the pie surrounded by multiple arcs represent polyfunctional cells. The pie arc legend shows IFN-**γ** in red, IL-17 in blue, IL-22 in green, and TNF-α in purple. p values were computed using the SPICE permutation test (25). Th17 cells show a significant decline in polyfunctional IL-17 responses from acute SIV to short-term cART, while Tc17 cells show an ongoing increase in monofunctional TNF- α responses during long-term cART.

Supplementary Figure 5 Clinical manifestations. **(A)** WBC counts **(B)** Body temperature, and (C) Body weight through the course of SIV infection and cART in the study animals.

Supplementary Figure 6 Kinetics of activation marker on various T cell subsets. HLA-DR expression on γδT cells (red bars), MAIT cells (black bars), CD161+CD4+ T cells (blue bars), and CD161+CD8+T cells (green bars) in peripheral blood during the course of SIV infection and cART. One-way ANOVA with Dunnett’s multiple comparisons test was used to determine significant differences from baseline. Asterisks indicate significant differences between time points (*p < 0.05; **p < 0.01; ***p < 0.001).
