# Supplementary figures and images for: Dysregulation of IL-17/IL-22 Effector Functions in Blood and Gut Mucosal Gamma Delta T Cells Correlates With Increase in Circulating Leaky Gut and Inflammatory Markers During cART-Treated Chronic SIV Infection in Macaques
Source: Front Immunol. 2021 Feb 25;12:647398. doi: 10.3389/fimmu.2021.647398 (PMC7946846; doi:10.3389/fimmu.2021.647398)

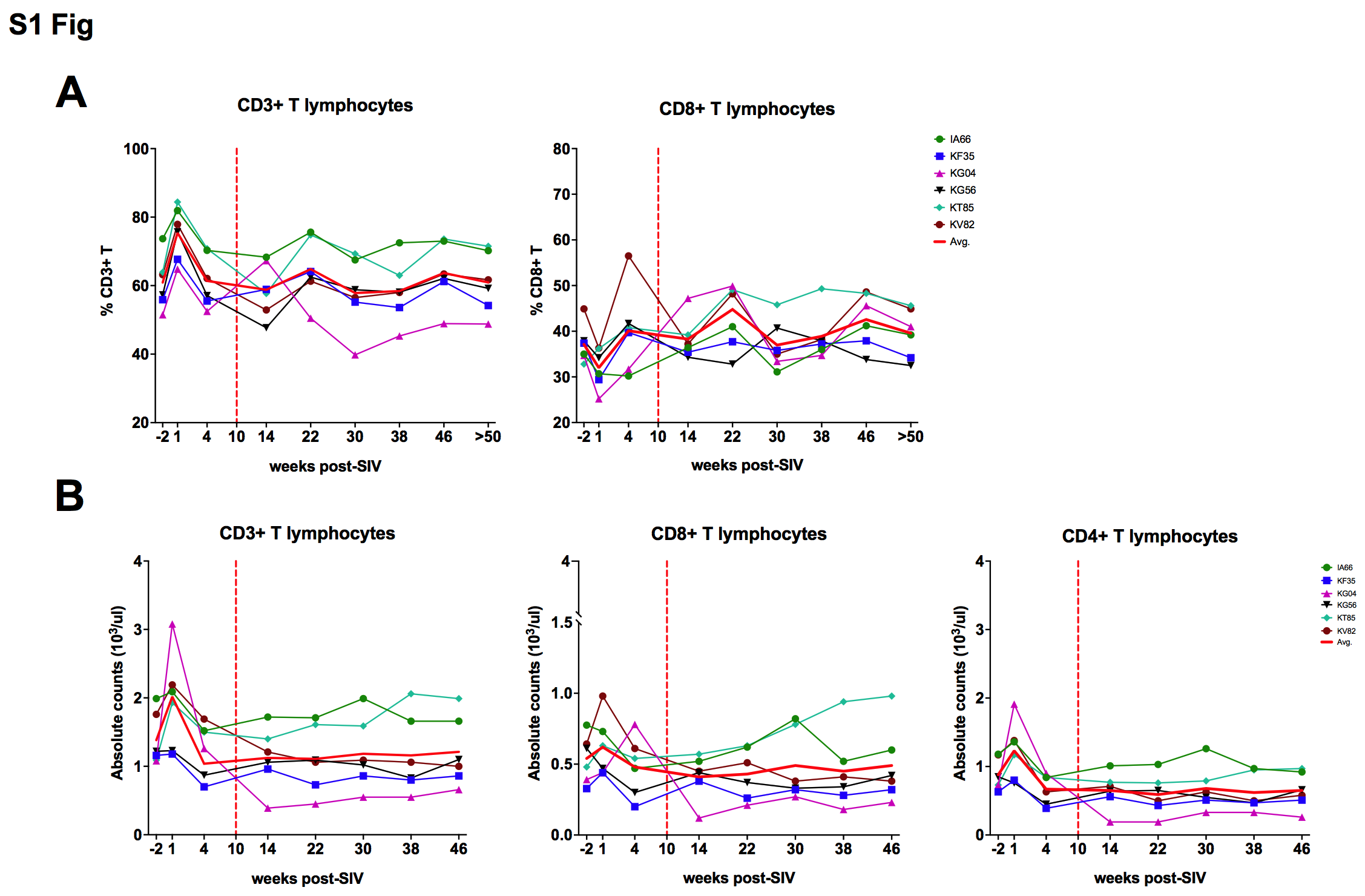

Supplement: Supplementary Figure 1 — Longitudinal assessment of percent and absolute number of T cells during the course of SIV infection and cART. (A) Percent CD3+ T lymphocytes and CD8+ T lymphocytes in PBMC at pre-SIV (week -2) and following SIV infection up to 50 weeks. (B) Absolute counts of total CD3+, CD8+ and CD4+ T lymphocytes per μl of peripheral blood at the indicated time points. [file Image_1.tif]

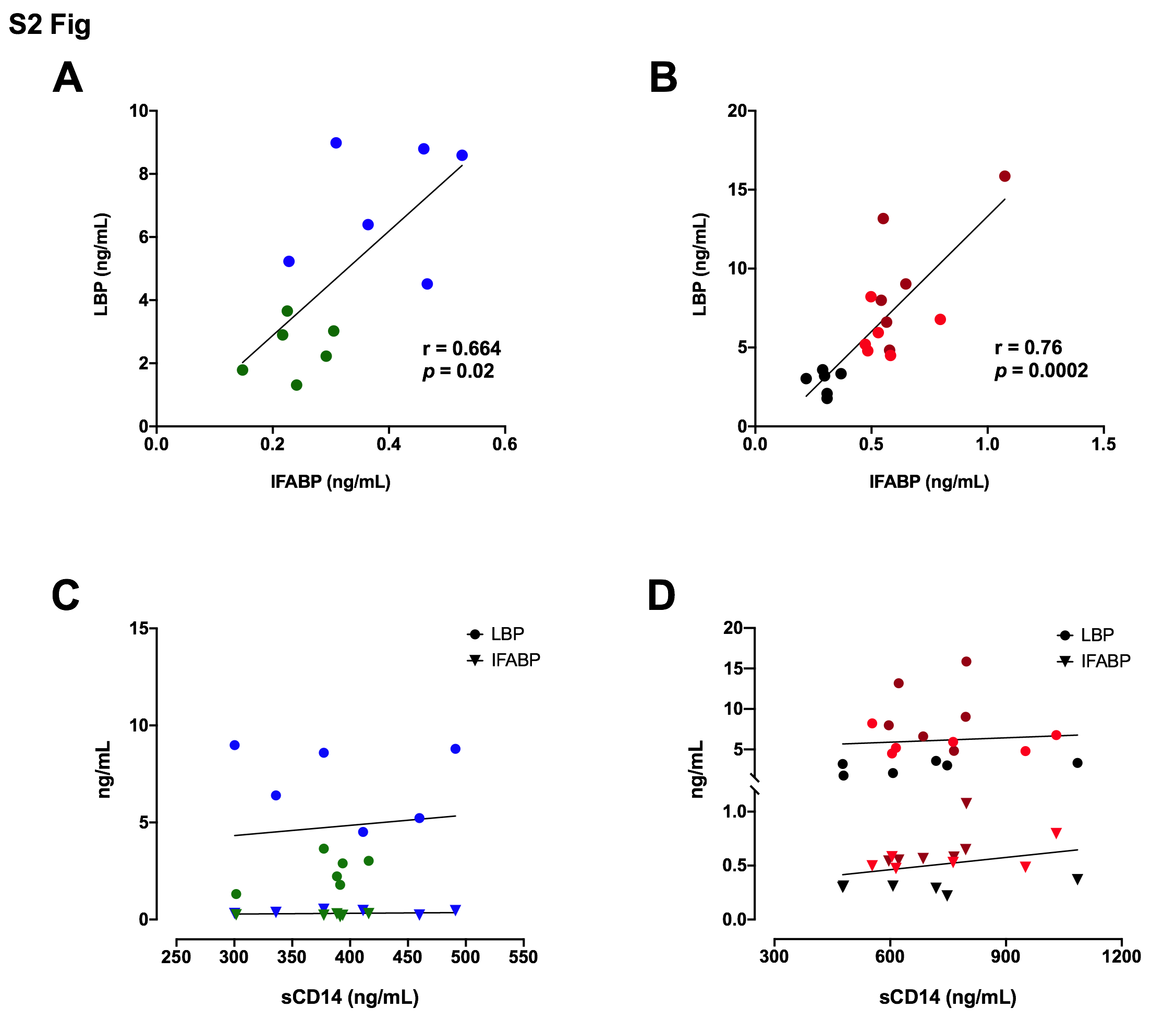

Supplement: Supplementary Figure 2 — Highly significant association of IFABP with LBP levels during long-term cART suppressed SIV infection. Spearman Rank correlation between plasma concentration of LBP and IFABP during: (A) acute SIV infection and early cART (1-month post-SIV: blue circles, 2-month cART: green circles), and (B) chronic cART-treated SIV infection (7-month: black circles, 8-month: crimson circles, and 11-month: red circles) in the study animals. Statistical significance (p values) and Spearman’s coefficient of rank correlation (r) are shown. Correlations of sCD14 with LBP and IFABP during: (C) acute SIV infection, and (d) chronic treated SIV infection showing no significant associations. [file Image_2.tif]

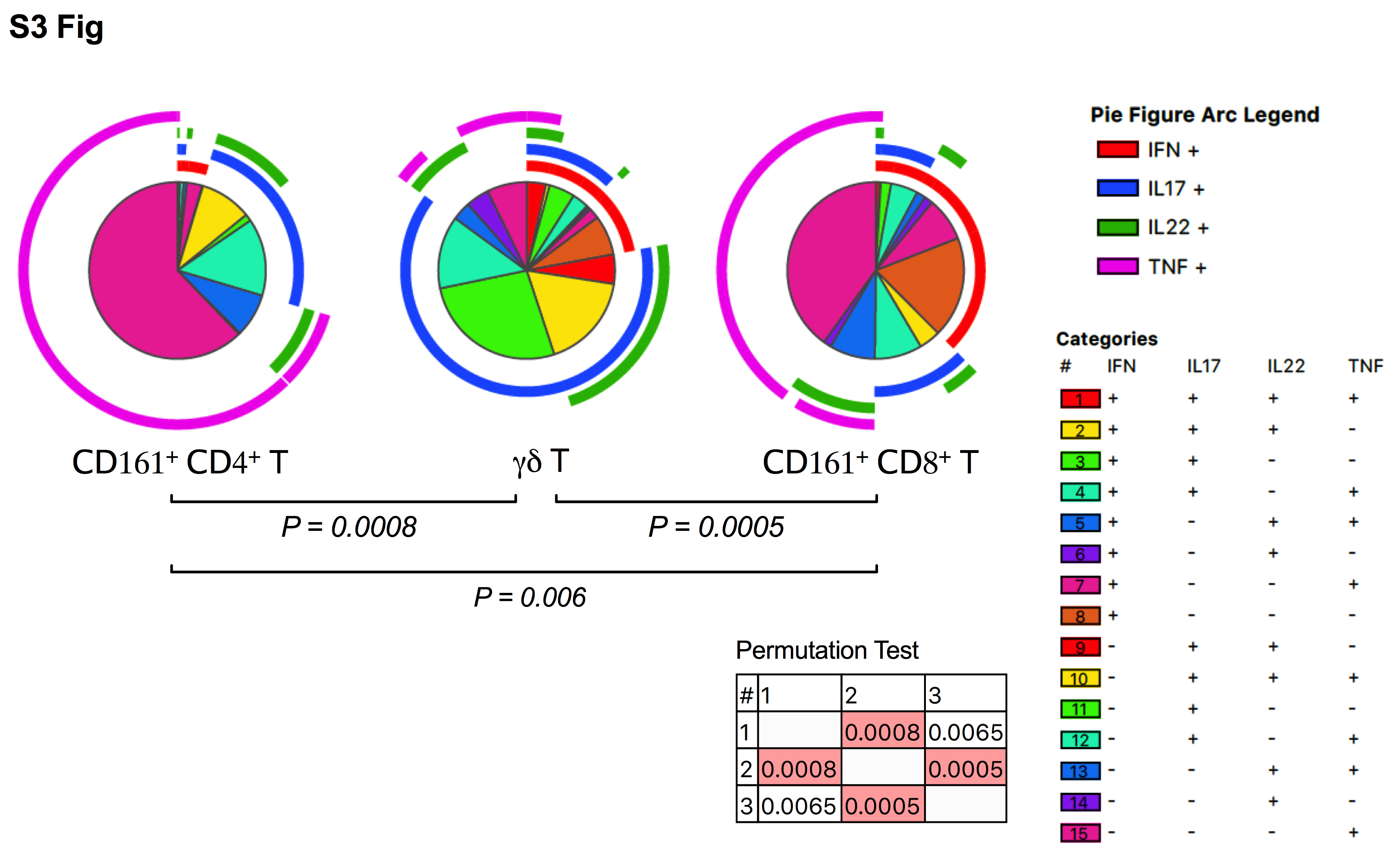

Supplement: Supplementary Figure 3 — γδT cells are a dominant source of polyfunctional IL-177/IL-22 responses in the gut. Pie charts comparing polyfunctional Th1/Th17 cytokine responses in Th17 (CD161+CD4+ T), γδT and Tc17 (CD161+CD4 = 8+ T) cells under steady state conditions. The pie charts represent the average frequencies of active cytokine-producing cells making every possible combination of IL-17, IL-22, TNF-α and IFN-γ. The segments within the pie chart denote populations producing different combinations of cytokines and are color coded. The arcs around the circumference indicate the particular cytokine produced by the proportion of cells that lie under the arc. Parts of the pie surrounded by multiple arcs represent polyfunctional cells. The pie arc legend shows IFN-γ in red, IL-17 in blue, IL-22 in green, and TNF-α in purple. p values were computed using the SPICE permutation test (25). [file Image_3.tif]

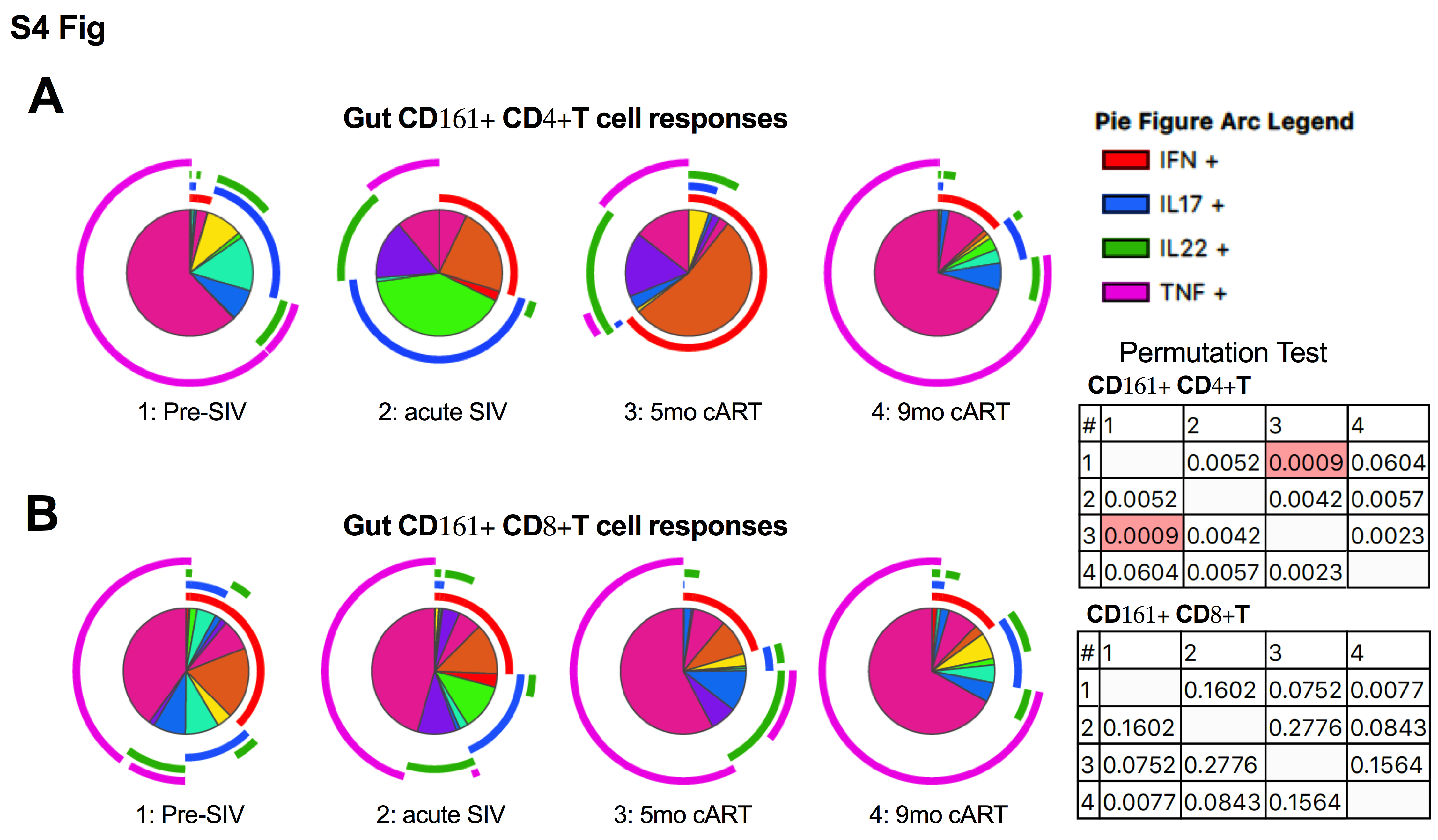

Supplement: Supplementary Figure 4 — Changes in rectal mucosal Th17 and Tc17 polyfunctional responses during long-term SIV suppression with cART. Pie charts comparing polyfunctional Th1/Th17 cytokine responses in (A) Th17 (CD161+CD4+ T), and (B) Tc17 (CD161+CD4 = 8+ T) cells at indicated time points. The pie charts represent the average frequencies of active cytokine-producing cells making every possible combination of IL-17, IL-22, TNF-α and IFN-γ. The segments within the pie chart denote populations producing different combinations of cytokines and are color coded. The arcs around the circumference indicate the particular cytokine produced by the proportion of cells that lie under the arc. Parts of the pie surrounded by multiple arcs represent polyfunctional cells. The pie arc legend shows IFN-γ in red, IL-17 in blue, IL-22 in green, and TNF-α in purple. p values were computed using the SPICE permutation test (25). Th17 cells show a significant decline in polyfunctional IL-17 responses from acute SIV to short-term cART, while Tc17 cells show an ongoing increase in monofunctional TNF- α responses during long-term cART. [file Image_4.tif]

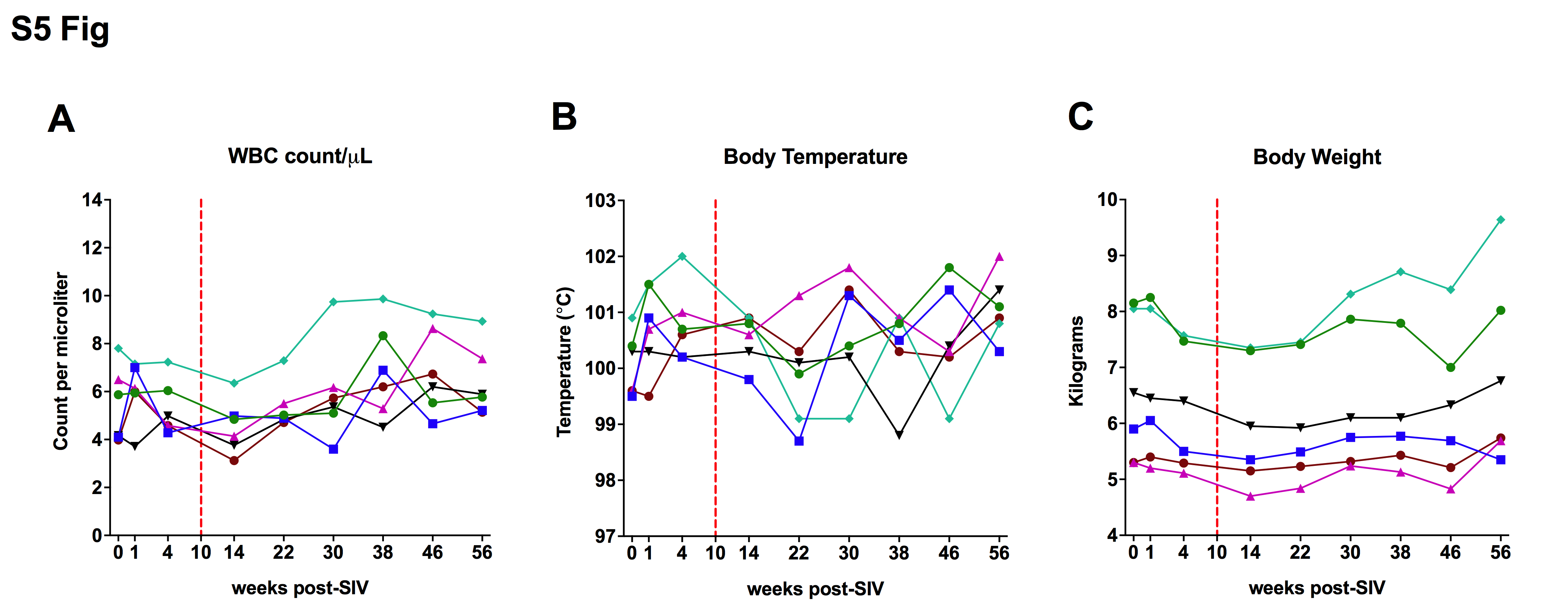

Supplement: Supplementary Figure 5 — Clinical manifestations. (A) WBC counts (B) Body temperature, and (C) Body weight through the course of SIV infection and cART in the study animals. [file Image_5.tiff]

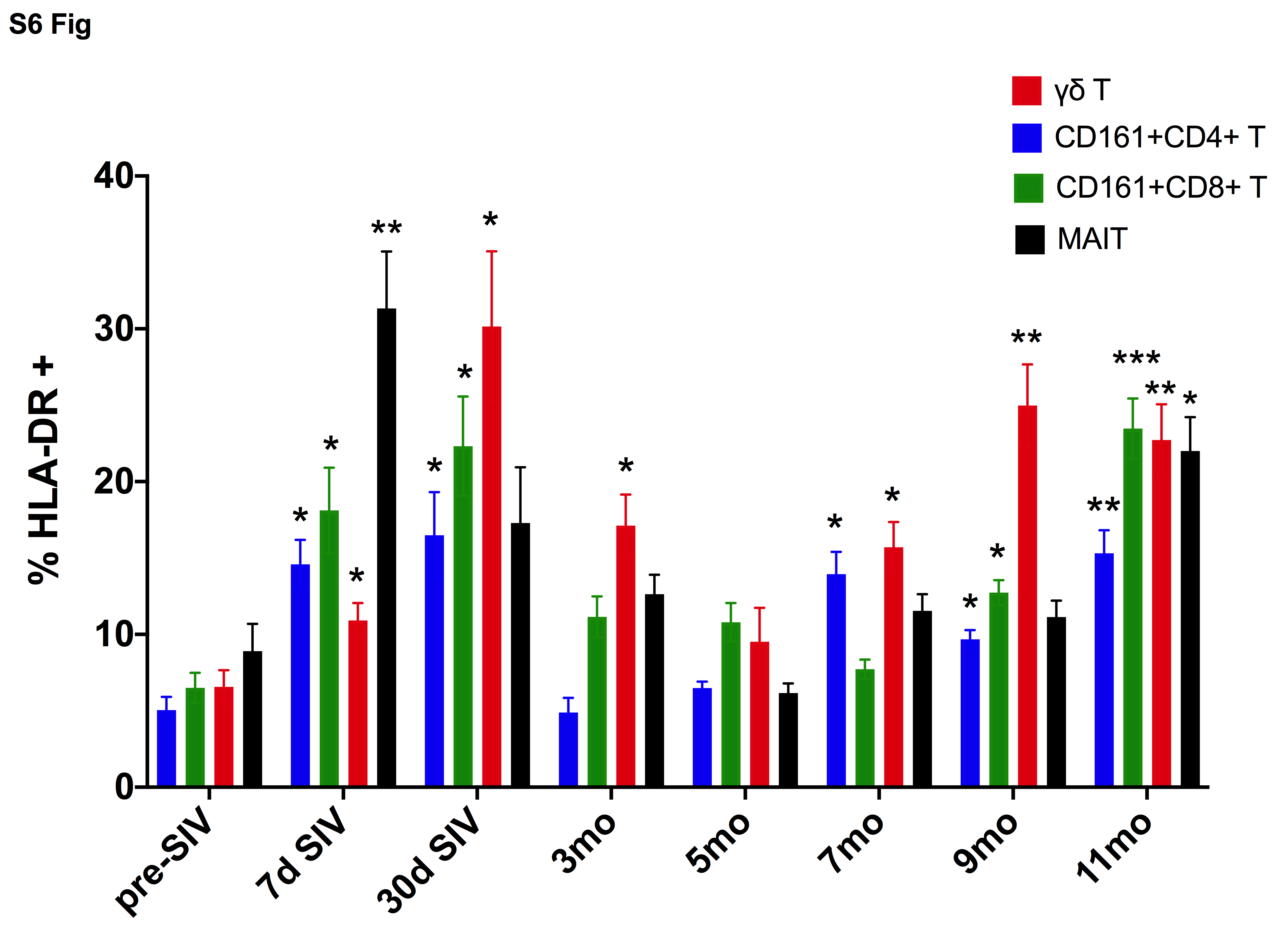

Supplement: Supplementary Figure 6 — Kinetics of activation marker on various T cell subsets. HLA-DR expression on γδT cells (red bars), MAIT cells (black bars), CD161+CD4+ T cells (blue bars), and CD161+CD8+T cells (green bars) in peripheral blood during the course of SIV infection and cART. One-way ANOVA with Dunnett’s multiple comparisons test was used to determine significant differences from baseline. Asterisks indicate significant differences between time points (*p < 0.05; **p < 0.01; ***p < 0.001). [file Image_6.tiff]
